# A Review on the Antidiabetic Properties of *Moringa oleifera* Extracts: Focusing on Oxidative Stress and Inflammation as Main Therapeutic Targets

**DOI:** 10.3389/fphar.2022.940572

**Published:** 2022-07-11

**Authors:** Fikile T. Mthiyane, Phiwayinkosi V. Dludla, Khanyisani Ziqubu, Sinenhlanhla X. H. Mthembu, Ndivhuwo Muvhulawa, Nokulunga Hlengwa, Bongani B. Nkambule, Sithandiwe E. Mazibuko-Mbeje

**Affiliations:** ^1^ Department of Biochemistry, North-West University, Mafikeng, South Africa; ^2^ Biomedical Research and Innovation Platform, South African Medical Research Council, Cape Town, South Africa; ^3^ Department of Biochemistry and Microbiology, University of Zululand, KwaDlangezwa, South Africa; ^4^ School of Laboratory Medicine and Medical Sciences, University of KwaZulu-Natal, Durban, South Africa

**Keywords:** diabetes complications, oxidative stress, inflammation, moringa (*Moringa oleifera*), therapeutic targets

## Abstract

*Moringa oleifera* is one of the popular plants that have shown significant health benefits. Certainly, preclinical evidence (predominantly from animal models) summarized in the current review supports the beneficial effects of *Moringa oleifera* leaf extracts in combating the prominent characteristic features of diabetes mellitus. This includes effective control of blood glucose or insulin levels, enhancement of insulin tissue sensitivity, improvement of blood lipid profiles, and protecting against organ damage under sustained conditions of hyperglycemia. Interestingly, as major complications implicated in the progression of diabetes, including organ damage, *Moringa oleifera* leaf and seed extracts could efficiently block the detrimental effects of oxidative stress and inflammation in these preclinical models. Notably, these extracts (especially leaf extracts) showed enhanced effects in strengthening intracellular antioxidant defences like catalase, superoxide dismutase, and glutathione to lower lipid peroxidation products and reduce prominent pro-inflammatory markers such as tumor necrosis factor-α, interleukin (1L)-β, IL-6, monocyte chemoattractant protein-1 and nitric oxide synthase. From animal models of diabetes, the common and effective dose of leaf extracts of *Moringa oleifera* was 100–300 mg/kg, within the treatment duration of 2–8 weeks. Whereas supplementation with approximately 20 g leaf powder of *Moringa oleifera* for at least 2 weeks could improve postprandial blood glucose in subjects with prediabetes or diabetes. Although limited clinical studies have been conducted on the antidiabetic properties of *Moringa oleifera*, current findings provide an important platform for future research directed at developing this plant as a functional food to manage diabetic complications.

## Introduction

According to the World Health Organization, diabetes mellitus is amongst the top ten leading causes of mortality and morbidity around the world (World Health Organization, 2022). Diabetes is a metabolic disorder that is characterized by a state of hyperglycemia, that occurs alongside dysregulations in insulin levels and in some cases, it arises concurrently to overweight and obesity (International Diabetes Federation, 2021). Indeed, excessive body fat or obesity remains the major culprits in the development of type 2 diabetes (T2D), which is the predominant form of diabetes (International Diabetes Federation, 2021). The rapid increase in cases of diabetes mellitus, especially T2D, raises concerns, also highlighting an urgent need to investigate effective therapies to curb this disease (Ahmad et al., 2019). Accumulative research has focused on understanding the pathophysiological mechanisms implicated in the development of diabetes-associated complications, which is essential to discover effective therapies that can improve metabolic function and prevent multiple organ failure in those affected by this condition (King and Brownlee, 1996; Fowler, 2007; Wei et al., 2020).

As a prime example, inflammation and oxidative stress, which normally emerge as a result of an abnormal pro-inflammatory response, or an exacerbated production of free radical species are increasingly recognized as the key abnormalities implicated in the development and acceleration of diabetes-linked complications (King and Brownlee, 1996). Notably, oxidative stress is linked with the activation of protein kinase C (PKC), which is normally consistent with impaired insulin signaling and tissue damage in experimental models of diabetes (King and Brownlee, 1996). Importantly, this content supports the hypothesis by Randle and others (Randle et al., 1963) which stated that alteration in the uptake and metabolism of glucose and free fatty acids may be an instrumental process in the pathogenesis of insulin resistance, the major characteristic feature of T2D. Indeed, many diverse biochemical mechanisms, extending beyond inflammation and oxidative stress or activation of PKC, are implicated in the development of T2D (King and Brownlee, 1996).

Literature suggests that effective modulation of energy metabolism and insulin signaling through the regulation of AMP-activated protein kinase (AMPK) or phosphoinositide 3-kinase/protein kinase B (PI3K/AKT) pathways appears to reverse some devastating outcomes of diabetes (Long and Zierath, 2006; Huang et al., 2018; Mazibuko-Mbeje et al., 2018). In fact, plants and their related bioactive compounds are increasingly screened for their antidiabetic properties. Some natural plants have shown anti diabetic properties through lowering blood glucose and modulation of AMPK/PI3K/AKT pathways (Francini et al., 2019; Mazibuko-Mbeje et al., 2019; Costa et al., 2020). Consistently, our group continues to review literature on the impact of plants like *Camellia sinensis* and *Aspalathus linearis*, including prominent bioactive compounds from some of these plants such as gallic acid and isoorientin for their ameliorative effects against metabolic complications (Dludla et al., 2019; Ziqubu et al., 2020; Dludla et al., 2021).

*Moringa oleifera* is a medicinal plant that has gained a lot of interest for its diverse biological properties. Reviewed evidence indicates the biological capabilities of this plant expand to protecting against complications linked with heart disease, cancer, fatty liver, and diabetes mellitus (Paikra et al., 2017; Vergara-Jimenez et al., 2017; Abd Rani et al., 2018). For example, a previously published review supported the beneficial effects of the leaves of the *Moringa oleifera* in improving blood glucose control in experimental models of diabetes (Ahmad et al., 2019). Notably, this review indicated draw backs such as the limited number of studies that have reported on the potential beneficial effects of this plant, including the fact that summarized literature was mainly conducted in animals, through *in vitro* and *in vivo* preclinical models. Nevertheless, while such information already affirms the hypoglycaemic potential of this medicinal plant, data regarding the prominent biochemical mechanisms implicated in the antidiabetic effects of *Moringa oleifera* have not been critically reviewed. Recently, Louisa and others supported the potential benefits of *Moringa oleifera* in cardiovascular or metabolic disorders, mainly by ameliorating the undesired pro-inflammatory response and inhibiting oxidative stress by mediating molecular mechanisms such as hindering nuclear factor kappa B (NF-κB) translocation or enhancing the antioxidant response of nuclear factor-erythroid factor 2-related factor 2 (Nrf2) in different preclinical models (Louisa et al., 2022). Thus, there is a need to better understand such intracellular responses of *Moringa oleifera* within a setting of diabetes or in related metabolic complications. The current study provides a brief overview on *Moringa oleifera* as medicinal plant, followed by its therapeutic mechanisms in controlling diverse diabetic complications. Mostly focusing on understanding the modulatory effects of this medicinal plant in mechanisms of inflammation and oxidative stress in a diabetic state.

This current review includes evidence that was obtained from a search done (from inception until end of December 2021) on major search engines such as PubMed, Google Scholar and ScienceDirect. The systematic search was conducted using the following Medical Subject Heading (MeSH) terms “*Moringa oleifera*”, “diabetes mellitus”, “glucose metabolism”, “insulin resistance”, “oxidative stress”, and “inflammation” as well as relevant synonyms. EndNote20 desktop software (Elsevier, Amsterdam, Netherlands) was used for references and identification of duplicated studies. Preclinical and clinical studies reporting on the mechanisms of *Moringa oleifera* in diabetes models and related metabolic syndrome was included in this review. However, review papers, and encyclopaedias were excluded but screened for primary findings. Notably, critical information related to the portion (part) of the plant that was assessed, as well as effective therapeutic dose and an experimental model used for the investigation is provided to better understand the potential benefits of *Moringa oleifera*.

## An Overview of *Moringa oleifera* and Its Diverse Biological Properties

**FIGURE 1:**
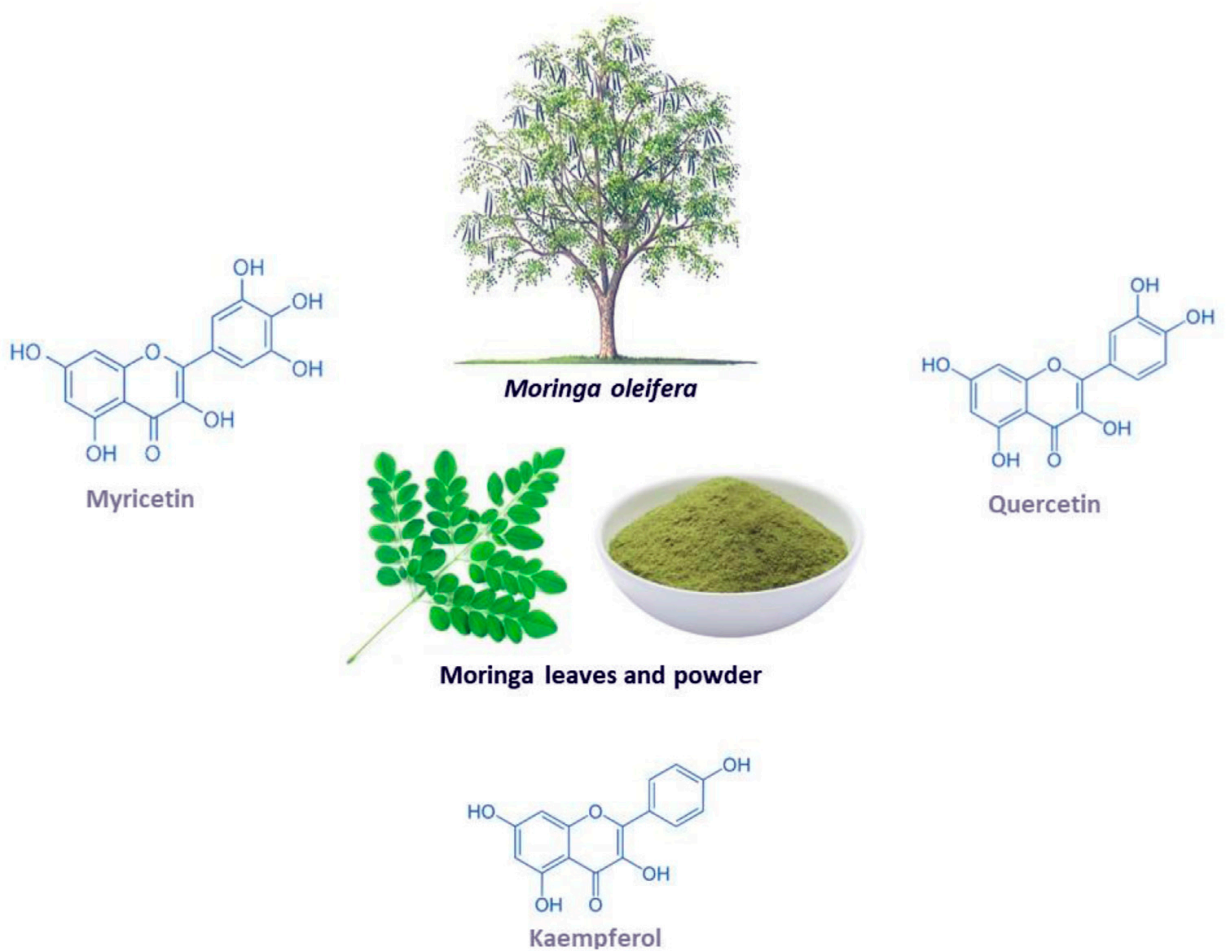
The *Moringa oleifera* plant, including the chemical structures of some of its major flavonoids myricetin, quercetin and kaempferol.

*Moringa oleifera* (shown in Figure 1) is a fast-growing tree that is classified as a vegetable that also serves as a medicinal plant (Gopalakrishnan et al., 2016; Trigo et al., 2020). This miracle tree originates from the sub-Himalayan parts of India, and it can be grown in both tropical and subtropical regions and is able to withstand droughts and mild frosty weather, hence it can be cultivated anywhere in the world (Gopalakrishnan et al., 2016)*.* This plant has gained medical and socioeconomic popularity because it has shown great health benefit and it is easy to cultivate (Alegbeleye, 2018; Zhu et al., 2020). Traditionally, it is applied in diets to maintain healthy skin and it has also been used as a decoction to relieve stress and provide energy (Mishra et al., 2011; Kumar et al., 2018). All the parts of the plant can be utilized in a diet or as medicine since they are rich in minerals, proteins, vitamins, polyphenols, flavonoids, glucosinolates, isothiocyanates, alkaloids (Gopalakrishnan et al., 2016; Trigo et al., 2020)*.* For example, the leaves can be eaten raw, dried or taken as an infusion of an aqueous extract, while the bark is boiled in water or soaked in alcohol to make drinks and infusions that help with toothaches, stomach aches, the same is done to the roots (Leone et al., 2015). Furthermore, the leaves are utilized the most for medicinal purposes and they are a great source of prominent anti-inflammatory and antioxidant flavonoids, namely myricetin, quercetin and kaempferol (Vergara-Jimenez et al., 2017). Interesting, these bioactive compounds are known to contain potential anticancer, hypolipidemic, hypotensive and antidiabetic properties, antioxidant and anti-inflammatory (Vergara-Jimenez et al., 2017). Other documented uses for this medicinal plant include its application as a diuretic, a testosterone stimulant, an antifungal and as an antibacterial (Mishra et al., 2011; Kumar et al., 2018). It can also be used to relieve a sore throat and symptoms of influenza, or as an anti-inflammatory agent (Mishra et al., 2011). Interestingly, evidence has grown that *Moringa oleifera* contains hypoglycemic effects in diabetic animal models, including its associated complications such as oxidative stress and inflammation (Balakrishnan et al., 2019; Chin et al., 2019; Bao et al., 2020).

## Oxidative Stress and Inflammation as Prominent Mechanisms Involved in Diabetes-Induced Complications

**FIGURE 2:**
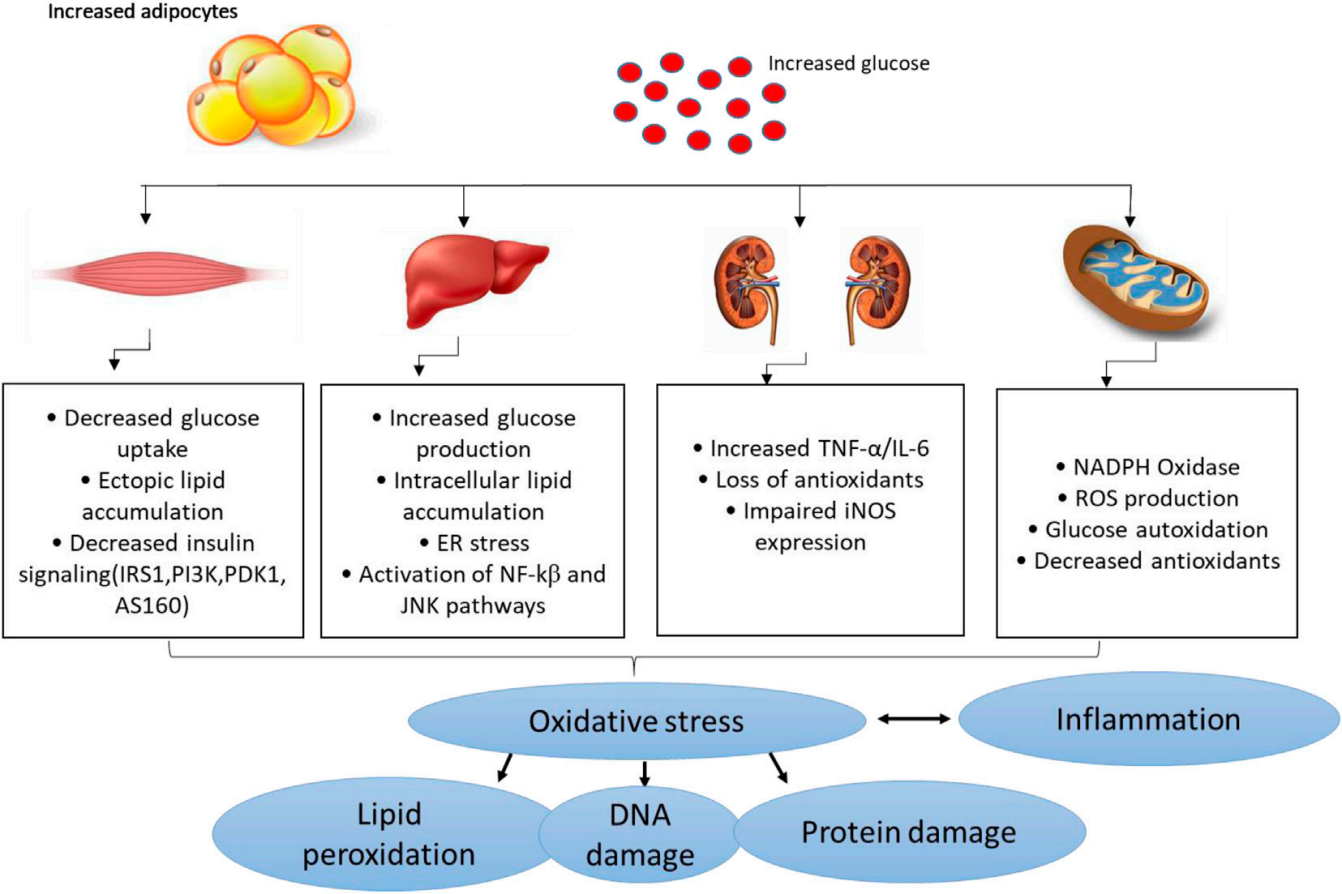
An overview of pathological mechanisms implicated in the development of diabetes mellitus or related metabolic complications. Briefly, overnutrition (which may be characterized by increased adipocyte size) and consistent increased levels of glucose (a state of hyperglycemia) may induce detrimental effects in major organs of the body including the skeletal muscle, liver, and kidneys, and thus aggravate metabolic complications through enhanced oxidative stress and exacerbated inflammation. This consequence is predominantly characterized by impaired glucose homeostasis/insulin signaling, ectopic lipid accumulation, mitochondrial dysfunction, endoplasmic reticulum (ER) stress insufficient or decreased antioxidant responses/increased ROS (reactive oxygen species) production and altered actions of inducible nitric oxide synthase (iNOS) and lipid peroxidation/DNA damage. This may occur along with raised pro-inflammatory markers like tumor necrosis factor-alpha (TNF-α), like nuclear factor kappa β (NF-κβ), c-Jun N-terminal kinases (JNK) and interleukin-6 (IL-6).

Several pathophysiological mechanisms have been implicated in the aggravation of diabetes-related complications. For instance, individuals with T2D already present with the dyslipidemic feature which is normally characterised by the highly accumulation of lipids and these can easily be attached by free radicals to generate damaging oxidative products (Biswas et al., 2017:; Ito et al., 2019). This consequence is referred to as lipid peroxidation, and it remains as one of the vital parameters used to track the devastating outcomes of oxidative stress in conditions of diabetes or any related metabolic disease (Rahimi et al., 2005; Grotto et al., 2009; Augustine et al., 2021). Within the pathological state, free radicals can be generated through impaired mitochondrial function, or enhanced activities of some enzymes complexes such as NADPH oxidases, in a process like oxidative stress that is known to deplete intracellular antioxidant systems (Mittal et al., 2014; He et al., 2017). Generally, oxidative stress is generated as a disparity in the production of reactive oxygen species (ROS) or reactive nitrogen species, in comparison to counteractive activity of antioxidants in diabetes (Giacco and Brownlee, 2010). Some of the prominent free radical molecules include hydroxyl radical (•OH), superoxide anion (O_**2**_^**•-**^), peroxynitrite (ONOO^−^), and all these molecules are crucial for efficient metabolic process in a physiological state (Burgos-mor et al., 2019; Chandra et al., 2019). Also, individuals with T2D display classic signatures of oxidative stress by presenting significantly decreased levels of antioxidant mechanisms such as superoxide dismutase (SOD), catalase (CAT) and glutathione peroxidase (GPx), and heme oxygenawe-1 (HO-1) (Matough et al., 2012; Sharma et al., 2012; Dumanović et al., 2021). In diabetes or related metabolic complications, uncontrolled ROS can induce damage to the lipids, proteins, and nucleic acids which lead to impaired signaling mechanisms and activation of pro-inflammatory response (Burgos-mor et al., 2019; Kim et al., 2020). Figure 2 gives an overview of some of the pathophysiological mechanisms implicating the detrimental effects of oxidative stress and inflammation in conditions of diabetes or related metabolic complications.

Currently, both oxidative stress and inflammation have been subject to ongoing research to improve metabolic function in conditions of syndrome (Furman et al., 2019; Monserrat-Mesquida et al., 2020; Oria et al., 2020). Also, accumulative research has evaluated the use of dietary compounds with antioxidant and anti-inflammatory effects such as salvianolic acid, aspalathin and resveratrol in combination with common drugs like metformin to lower glucose as well as attenuate the detrimental effects of oxidative stress and inflammation (Frendo-Cumbo et al., 2016; Wu et al., 2016; Dludla et al., 2018). This has been especially important aspect uncover to improve the long-term protective effects of metformin. The latter is the first line drug for diabetes which works by lowering blood glucose, body weight and lipid levels in the body it also mediates the activation of the AMPK pathway. On the other hand, other antidiabetic drugs like the thiozonidediones have been used to manage T2D, and function by activating peroxisome proliferator activated receptor gamma (PPARγ) and mediate adipogenesis and the uptake of fatty acids in the adipocytes (Greenfield and Chisholm, 2004). This class of drugs improve insulin sensitivity by reducing the circulating fatty acids in the peripheral tissues and can control the production of hormones such as adiponectin to improve metabolic function (Greenfield and Chisholm, 2004). However, like metformin, thiozonidediones are known to present with various side effects (DeFronzo et al., 2016), and their long-term protects effects against deteriorated metabolic function is not proven. This fact, has created opportunities to evaluate alternative regimes, including medicinal plants like *Moringa oleifera* with antioxidant and anti-inflammatory for their capacity to improve metabolic function in conditions of T2D or metabolic syndrome (Rena et al., 2013; Yendapally et al., 2020). This is especially important since most medicinal plants and bioactive compounds are known to play a major role in cellular detoxifying mechanisms, especially in part activating Nrf2, the major antioxidant response element involved in the attenuation of oxidative stress and an undesired pro-inflammatory response in a disease state (Ma, 2013; Minhaj et al., 2016; Dludla et al., 2017b).

## The Potential *in Vitro* Antioxidant Properties of *Moringa oleifera*

Antioxidants are important substances that aid in eliminating oxidizing agents. Any imbalance of antioxidants caused by oxidative stress may lead to tissue damage (Kurutas, 2016). This may further prompt the disruption of lipids, membranes, nucleic acids and proteins which may further cause detrimental effect and metabolic complications (Phaniendra and Babu, 2015; Pizzino et al., 2017). For years, the first line of drugs for metabolic complications such as diabetes and related metabolic disorders have been metformin, thiazolidinediones and rosiglitazone but literature has proven that plant polyphenols and their bioactive compounds may potentially provide more efficacy in alleviating diabetes, especially through targeting oxidative stress and inflammation to promote human health (Marimoutou et al., 2015; Singh et al., 2016; Taïl et al., 2020; Do et al., 2021). For example, a study showed that *Moringa oleifera* has great scavenging activity, as measured through the DPPH (1,1-diphenyl-2-picrylhydrazyl (DPPH)-2,2-diphenyl-1-picrylhydrazyl) and ABTS (2,2′-azino-bis(3-ethylbenzothiazoline-6-sulfonic acid) (Pakade and Chimuka, 1996). However, these results were purely relevant to its potential antioxidant potential, meaning that additional studies, making use of established preclinical and clinical models of diabetes, were still required to confirm the efficacy of this plant. Congruently, the below preclinical and clinical evidence discusses the therapeutic potential of this plant to reduce limit pathological features of oxidative stress and inflammation to alleviate complications linked with diabetes and related metabolic complications, without causing any adverse complications.

## *Moringa oleifera* Extracts Improve Markers of Oxidative Stress and Inflammation in Preclinical Models of T1D

Type 1 diabetes mellitus represents approximately 10% of all diagnosed cases of diabetes mellitus, with abnormally increased glucose levels “a state of hyperglycemia” being the major culprit implicated in the detrimental effects associated with this condition (Internationa Diabetes Federation, 2021). Generally accepted as an autoimmune disease that is categorized by immune-mediated damage to pancreatic β-cells, a persistent state of hyperglycemia is accredited for the damaging effects on major organs of the body in those with T1D (Roep et al., 2021). Accordingly, successful establishment of experimental models of T1D, characterized by chronic hyperglycemia, have been predominantly accomplished by employing chemicals that destroy the activity of pancreatic β-cells, triggering impaired insulin secretion (Kottaisamy et al., 2021). Persistent/sustained hyperglycemia is perhaps the main consequence responsible for major organ damage, especially through destructive mechanisms involving activation of oxidative stress and promoting a pro-inflammatory response (Giacco and Brownlee, 2010). Thus far, different animals, mostly rodents have been administering different chemicals such as streptozotocin and alloxan to generate preclinical models of T1D (Kottaisamy et al., 2021). Despite their usefulness in understanding the pathogenesis of T1D, these experimental models have become relevant for screening novel drugs for their potential antidiabetic properties (King, 2012). In fact, besides their potential capacity to reduce the abnormally elevated levels of glucose, increasing research has actively screened novel therapies for their ameliorative effects against oxidative stress and inflammation to alleviate organ damage within a state of T1D (Mima, 2013; Mokgalaboni et al., 2021a; Perreault et al., 2021). This has especially been relevant for plant sources like *Moringa oleifera*, with known antioxidant and anti-inflammatory properties (Xu et al., 2019).

Evidence summarized in Table 1 reports on the impact of *Moringa oleifera* extracts on modulating markers of oxidative stress and inflammation in preclinical models of T1D. Importantly, most of these findings indicate that chemical exposure to STZ and alloxan, followed by a state of hyperglycemia, remains a principal method used to induce T1D in these animals. Consequently, most studies showed that *Moringa oleifera* extracts (at varied doses between 100 and 300 mg/kg) could effectively ameliorate hyperglycemia, when used for a period starting from 2 weeks (Tuorkey, 2016; Oboh et al., 2018), to an average time of 6-weeks (Omodanisi et al., 2017a; Omodanisi et al., 2017b), or even in treatments lasting 8-weeks (Yassa and Tohamy, 2014; Aju et al., 2020). Interestingly, treatment with *Moringa oleifera* leaf extracts proved effective in wound healing and tissue regeneration in animals exposed to sustained levels of hyperglycemia, when used for an estimated time of 3 weeks (Muhammad et al., 2016; Azevedo et al., 2018). In addition to wound healing properties, the extracts *Moringa oleifera* showed enhanced protective effects against damage to various organs, including the liver and kidneys, in these preclinical models of T1D (Abd Eldaim et al., 2017; Omodanisi et al., 2017a; Oguntibeju, 2019; Oldoni et al., 2021). The antidiabetic properties of these extracts extend to preventing cognitive or erectile dysfunction in rats, by mainly reducing the activities of enzymes like acetylcholinesterase, angiotensin-I converting enzyme and butyrylcholinesterase (Oboh et al., 2018; Oyeleye et al., 2021). Apparently, the ameliorative effects against oxidative stress or undesired pro-inflammatory response remain the predominant mechanisms by *Moringa oleifera* extracts protect against complications of T1D in preclinical (animal) models.

**TABLE 1:** Studies on the effect of *Moringa oleifera* extracts targeting markers of oxidative stress and inflammation in preclinical models of type 1 diabetes.

Author, year	Experimental model, effective dose and intervention period	Experimental outcome
Jaiswal et al. (2013)	Streptozotocin (STZ)-induced diabetes in Wistar rats treated with 200 mg/kg *Moringa oleifera* leaf extract for 3 weeks	Ameliorated oxidative stress by significantly increasing the antioxidant activity of superoxide dismutase (SOD), glutathione S-transferase (GST) and catalase (CAT) while decreasing the lipid peroxide levels
Yassa and Tohamy, (2014)	STZ-induced diabetes in Sprague Dawley rats treated with 200 mg/kg *Moringa oleifera* extract for 8 weeks	Lowered fasting plasma glucose (FPG) levels, reversed pancreatic damage, while also enhancing glutathione (GSH) and reducing malondialdehyde (MDA) pancreatic concentrations
Al-Malki El Rabey, (2015)	STZ-induced diabetes in albino rats were treated with 50 and 100 mg/kg with *Moringa oleifera* seed extract for 4 weeks	Decreased FPG, and increased the concentration of antioxidants like SOD, CAT and GSH in serum and kidney. Moreover, treatment decreased the concentration of interleukin (IL)-6 and lipid peroxides (MDA) in the serum and kidney tissue homogenate
Muhammad et al. (2016)	STZ-nicotinamide induced diabetes in Wistar rats treated with 0.5,1 and 2% w/w *Moringa oleifera* leaf aqueous fractions for 3 weeks	Decreased wound size under sustained hyperglycemic condition and improved wound contraction, and tissue regeneration. This was associated with reduced inflammatory mediator such as tumor necrosis factor alpha (TNF-α), IL-1β, IL-6, cyclooxygenase-2 (COX-2), nitric oxide synthase (iNOS) and upregulation of an angiogenic marker vascular endothelial growth factor in wound tissue
Tuorkey, (2016)	Alloxan-induced diabetes albino mice treated with 100 mg/kg of *Moringa oleifera* aqueous extract 2 weeks	Significantly decreased FPG and plasma insulin levels, concomitant to reversing insulin resistance. Total antioxidant capacity increased while the levels of creatinine and urea significantly declined. While cluster of differentiation (CD)44 was not changed, CD69 and interferon gamma I (NF-γ) were increased by treatment
Abd Eldaim et al. (2017)	Alloxan-induced diabetes in Wistar rats treated with 250 mg/kg *Moringa oleifera* leaf extract for 2.5 weeks	Prevented hepatic damage and normalized the reduced hepatic levels of glutathione (GSH), as well as the activities of SOD and CAT, while also reducing blood glucose levels, hepatic lipid peroxidation
Omodanisi et al. (2017a)	STZ-induced diabetes in Wistar rats treated with 250 mg/kg *Moringa oleifera* aqueous extract for 6 weeks	Reduced hepatic enzyme markers and normalized lipid profile parameters, while enhancing antioxidant capacity and alleviating inflammatory biomarkers of the liver. Specifically, reduced levels of MDA and increased endogenous antioxidants (SOD, CAT, GSH, GPx), while decreased inflammatory cytokines (IL-1α, IL-6, IL-12, IL-18, TNF-α) and (chemokine: MCP-1) in the serum, liver; kidney and erythrocytes
Omodanisi et al. (2017b)	STZ-induced diabetes in Wistar rats treated with 250 mg/kg *Moringa oleifera* leaf extract for 6 weeks	Reduced FPG and biomarkers of oxidative stress and inflammation. Specifically, reduced the level of lipid peroxidation (MDA), and improved antioxidant such as CAT, SOD, GSH, glutathione peroxidase (GPx), whilst decreasing pro-inflammatory makers such as tumor necrosis factor-alpha (TNF-α) and IL-6
Raafat and Hdaib, (2017)	Alloxan-induced diabetes albino rats treated with 250 mg/kg *Moringa oleifera* leaf extract for 2.5 weeks	Reduced FPG and hindered lipid peroxidation, whilst increasing hepatic GSH levels, as well as the activities of SOD and CAT, and the gene expression of glycogen synthase while reducing pyruvate carboxylase caspase 3 gene expression
	Alloxan-induced diabetes Swiss-Webster mice treated with 40,60 and 80 μg/ml *Moringa oleifera* seed extract for 8 weeks	Decreased FPG and inhibited alpha glucosidase activity. Serum insulin levels and serum CAT levels were significantly increased whilst lipid peroxidation and glycated hemoglobin (HbA1C) were decreased
Alejandra Sánchez-Muñoz et al. (2018)	STZ-induced diabetes in Wistar rats treated with 200 mg/kg *Moringa oleifera* leaf extract for 3 weeks	Ameliorated oxidative stress-induced modification in liver mitochondria, in part by improving mitochondrial respiration, as well as enhancing the antioxidant levels of GSH, glutathione reductase and heme oygenase-1 (HO-1) activity, which decreasing lipid peroxidation (MDA) and production of reactive oxygen species
Azevedo et al. (2018)	STZ-induced diabetes in Wistar adult rats were treated with 100 mg/kg of *Moringa oleifera* leaf extract for 3 weeks	Displayed wound healing properties and significantly reduced glycemia accompanied by a decreased in pro-inflammatory markers such as TNF-α, IL-ꞵ and IL-6 in the serum
Oboh et al. (2018)	STZ-induced diabetes in Wistar rats treated with 2 and 4% *Moringa oleifera* leaf and seed extracts for 2 weeks	Both extracts reduced FPG, prevented cognitive dysfunction-induced by chronic hyperglycemia by reducing the activities of acetylcholinesterase, angiotensin-I converting enzyme and butyrylcholinesterase. This was concomitant to the increase in antioxidant molecules such as CAT, GST and GPx, as well as a decrease in lipid peroxidation (MDA) level in the brain
Aju et al. (2019)	STZ-induced diabetes in Sprague-Dawley rats treated with 300 mg/kg body weight leaf (methanol) extract for 8.5 weeks	Significantly decreased FPG and glycated hemoglobin but increased plasma insulin levels. The antioxidant enzymes like SOD, CAT, GPx and glutathione-reductase and non-enzymatic antioxidant GSH were increased causing a decrease in hydroperoxides, conjugated dienes and lipid peroxidation
Oguntibeju et al. (2020)	STZ-induced diabetes Wistar rats treated with 250 mg/kg leaf extract of *Moringa oleifera* for 6 weeks	Reduced nephrotoxic and hepatotoxic damage evident by a decrease in serum creatinine, albumin and bilirubin. Likewise, the inflammatory cytokines interleukin (IL)-1α, IL-12 and IL-18, and apoptotic markers caspase 3, caspase 9, B-cell lymphoma 2(BCL-2), NF-κβ, and p53 were decreased
Sierra-Campos et al. (2020)	Alloxan-induced diabetes in Wistar rats treated with 200 mg/kg leaf extract of *Moringa oleifera* for 3 weeks	Displayed antidiabetic effects by increasing the levels of serum paraoxonase 1 and liver cytosolic CAT
Oldoni et al. (2021)	STZ-induced diabetes in Wistar rats treated with 500 mg/kg crude leaf extract of *Moringa oleifera* (hydroalcoholic extract was produced by using ethanol: water (80:20 v/v)) for 6.5 weeks	Reduced FPG and protected against oxidative damage in liver and kidney by enhancing endogenous antioxidant defenses such as CAT, GST and non-protein thiol groups, while reducing lipid peroxidation in these tissues
Oyeleye et al. (2021)	STZ-induced diabetes in Wistar rats treated with 2 and 4% of *Moringa oleifera* leaf and seed extracts for 2 weeks	Both extracts reversed diabetes-induced erectile dysfunction by reducing FPG, as well as blocking lipid peroxidation by decreasing thiobarbituric acid reactive species (TBARS) levels. Treatments also attenuated the activity of phosphodiesterase type 5 (PDE-5) and arginase but improved the levels of nitric oxide

For instance, through blockade of lipid peroxidation as well as by reinforcing intracellular antioxidant capacity, as demonstrated by reduced levels of peroxidation products like MDA/thiobarbituric acid reactive species (TBARS) and elevated antioxidant defences such as SOD, GSH, GST and CAT, *Moringa oleifera* extracts showed enhanced effects in protecting against the detrimental effects of oxidative stress in preclinical models of T1D (Jaiswal et al., 2013; Yassa and Tohamy, 2014; Al-Malki and El Rabey, 2015; Raafat and Hdaib, 2017). In support of this mechanistic insight, it has long been established that induction of diabetes in rats with STZ or alloxan favors uncontrolled availability of lipid peroxidation products, while consequently suppressing the intracellular antioxidant defences (Maritim et al., 2003; Davì et al., 2005). This process prompts excess free radical production, as also observed in patients with T1D (Domínguez et al., 1998), while the accompanied hyperglycemic state may directly contribute to oxidative stress-induced organ damage (Maritim et al., 2003). Besides the harmful effects associated with lipid peroxidation, evidence summarized in Table 1 indicates that treatment with *Moringa oleifera* extracts for 3 weeks remains effective in targeting other sources of oxidative stress like the mitochondria to ease complications linked with T1D. In actual fact, Alejandra Sánchez-Muñoz and others showed that a leaf extract of this plant improved mitochondrial respiration, while increasing levels of intracellular antioxidants like GSH, glutathione reductase and HO-1 activity, to reduce excess production of ROS liver cells of STZ-induced diabetic rats (Alejandra Sánchez-Muñoz et al., 2018). Generally, these results are of interest as many studies indicate that mitochondria remain one of the major therapeutic targets to ameliorate hyperglycemia-induced oxidative damage (Giacco and Brownlee, 2010; Dludla et al., 2017a; Teodoro et al., 2018).

Consistent with attenuating the destructive effects of oxidative stress, presented evidence showed that *Moringa oleifera* extracts could effectively reduce the elevated levels of pro-inflammatory mediators such as tumor necrosis factor alpha (TNF-α), IL-1β, IL-6, cyclooxygenase-2 (COX-2), nitric oxide synthase (iNOS), and chemokine (MCP-1) to protect against exacerbated inflammation, under sustained conditions of hyperglycemia (Muhammad et al., 2016; Omodanisi et al., 2017a; Azevedo et al., 2018). Significantly, such positive effects were associated with reduced nephrotoxic and hepatotoxic damage (Oguntibeju, 2019), including upregulation of an angiogenic marker vascular endothelial growth factor (VEGF) to protect against hyperglycemia-induced wound injury in a preclinical model of T1D (Muhammad et al., 2016). This is an essential result since tenacious hyperglycemia, seen in T1D, is already known to mediate iNOS induction, leading to the activation of protein kinase enzymes such as PKC/c-Jun N-terminal kinases (JNKs)/mitogen-activated protein kinase (MAPK) to propagate the detrimental effects of inflammation (Giacco and Brownlee, 2010). Notably, activation of such pro-inflammatory mechanisms can directly cause excess generation of oxidation products that precede the onset of atherosclerosis and endothelial dysfunction, which are major risk factors for the development of cardiovascular diseases (Rose et al., 2010; Mokgalaboni et al., 2020). Although there was limited information on its cardioprotective effects, much evidence suggests *Moringa oleifera* extracts can significantly decrease pro-inflammatory and apoptotic markers such as TNF-α, IL-1β, IL-6, NF-κβ, caspase 3, caspase 9, and tumor protein (p53) to alleviate the detrimental effects of hyperglycemia in preclinical models of T1D (Muhammad et al., 2016; Omodanisi et al., 2017a; Azevedo et al., 2018; Oguntibeju, 2019). Overall, summarized evidence supports the beneficial effects of *Moringa oleifera* in lowering hyperglycemia in addition to ameliorating the detrimental effects of oxidative stress and inflammation in preclinical (animal) models of T1D. Some other takeaways from the current results indicate that most studies observed therapeutic effects when using doses between 100 and 300 mg/kg (Al-Malki and El Rabey, 2015; Tuorkey, 2016; Alejandra Sánchez-Muñoz et al., 2018; Azevedo et al., 2018), with an average dose of 250 mg/kg commonly exploited (Abd Eldaim et al., 2017; Omodanisi et al., 2017a; Omodanisi et al., 2017b; Raafat and Hdaib, 2017). Also, most studies reported on the use of leaf extracts over seed extracts of this plant (Table 1). This could be supported by available evidence already supporting the strong antioxidant properties of leaf extracts of over seed extracts (Ilyas et al., 2015; Saini et al., 2016).

## *Moringa oleifera* Extracts Improve Markers of Oxidative Stress and Inflammation in Preclinical Models of T2D

Type 2 diabetes, remains the major form of diabetes, contributing to approximately 90% to all global cases of this condition, as regularly reported by the world leading health surveillance organizations (International Diabetes Federation, 2021). Modifiable risk factors, mostly involving sedentary lifestyle, taking place together with overnutrition are to blame for increased cases of T2D, as these factors cause overweight and obesity (Grundy, 2016). In such conditions, increased nutrient availability may drive excessive fat accumulation in key body areas such as the liver, skeletal muscle, blood circulation and heart muscle, leading to the development of pathological complications like non-alcoholic fatty liver disease, muscle wasting or sarcopenia and cardiovascular diseases (Grundy, 2016; Chait and den Hartigh, 2020). Just like in T1D, hyperglycemia remains the major pathological feature of T2D. Besides hyperglycemia, patients with T2D are known to present with insulin resistance and a cluster of other irregularities such as abnormal blood lipid profiles, as observed through aberrant levels of triglycerides, total cholesterol, and low-density lipoprotein cholesterol (Stanford and Goodyear, 2014; Oguntibeju, 2019). However, defects in insulin signaling or a state of insulin resistance has been seen as an early sign of T2D manifestation, which is likely to occur simultaneous to other metabolic dysregulations, including enhanced inflammatory signaling, generation of oxidative products and initiation of endoplasmic reticulum stress pathways (Muoio and Newgard, 2008). These are among the most explored pathological mechanisms in preclinical models of T2D. For example, rodents exposed to a high fat diet (HFD) or its combination with low dose STZ (Waterman et al., 2015; Jaja-Chimedza et al., 2018; Chin et al., 2019; Mohamed et al., 2019; El-Shehawi et al., 2021), as well as gene-defiant mice such as those considered leptin resistance (*db/db*) (Tang et al., 2017) are known to progressively develop T2D, including its complications involving oxidative stress and inflammation. This explains, the surge use of these preclinical models to test novel treatments against T2D.

Table 2 gives an overview of information on the antidiabetic properties of *Moringa oleifera* extracts, including its modulatory effects on markers of oxidative stress and inflammation in preclinical models of T2D. Most importantly, summarized evidence showed that these extracts were effective in reducing body weight, body fat mass and fasting plasma glucose levels, which are the major characteristic features of T2D (Tang et al., 2017; Jaja-Chimedza et al., 2018; El-Shehawi et al., 2021). Consistent with evidence summarized in Table 1, blocking hepatic lipid accumulation, in part through effective modulating the makers of oxidative stress and inflammation such as antioxidants like CAT, SOD, MDA content, uncoupling protein 2/3, TNF-α, 1L-β, IL-6, IL-2 and MCP-1 remains the major mechanism of action of these extracts (Joung et al., 2017; Mohamed et al., 2019). Some evidence showed these extracts could improve lipid profiles and reduce the expression of genes involved in energy metabolism or fat synthesis such as fatty-acid synthase, lipoprotein lipase, CCAAT-enhancer-binding protein homologous-α (C/EBPα), sterol regulatory element-binding protein 1c (SREBP1c), within the skeletal muscle (Joung et al., 2017; Tang et al., 2017). Partially indicating that *Moringa oleifera* extracts may be a potent remedy to decrease excess body fat or ameliorate complications linked with obesity, as reviewed elsewhere (Redha et al., 2021). Of further interest, some evidence indicated that *Moringa oleifera* extracts could target early pathological signs of T2D, such as improving glucose tolerance and insulin levels, while enhancing insulin sensitivity and glucose homeostasis in tissues of these preclinical models (Jaja-Chimedza et al., 2018; Mohamed et al., 2019). This hypothesis remains to be confirmed in other experimental models of T2D, however provides necessary information to guide future research.

**TABLE 2:** An overview of studies on the effects of *Moringa oleifera* extracts targeting markers of oxidative stress and inflammation in preclinical models of type 2 diabetes.

Author, year	Experimental model, effective dose and intervention period	Experimental outcome
Waterman et al. (2015)	High fat diet (HFD)-fed C57BL/6L mice treated with 5% *Moringa oleifera* concentrate (delivering 66 mg/kg/d of moringa isothiocyanates)	Improved glucose tolerance and insulin signaling and did not develop fatty liver disease. Treatment also reduced plasma insulin, leptin, resistin, cholesterol, interleukin (IL)-1β, tumor necrosis factor alpha (TNF-α), and lowered hepatic glucose-6-phosphatase expression
Joung et al. (2017)	HFD-induced glucose intolerance C57BL/6 mice treated with 250 mg/kg *Moringa oleifera* leaf extract for 10 weeks	Did not affect body weights but reduced hepatic lipid accumulation. Also, reduced HFD-induced endoplasmic reticulum stress, oxidative stress, and lipotoxicity in quadriceps muscles. Reduced the expression of genes involved in energy metabolism such as fatty-acid synthase, lipoprotein lipase, CCAAT-enhancer-binding protein homologous-α (C/EBPα), sterol regulatory element-binding protein 1c (SREBP1c), within the skeletal muscle. Oxidative and inflammatory markers such as uncoupling protein 2/3, TNF-α, 1L-β, IL-6, IL-2 and monocyte chemoattractant protein-1 (MCP-1) were improved
Tang et al. (2017)	Type 2 diabetic (*db/db*) mice treated with 150 mg/kg *Moringa oleifera* leaf ethanolic extract for 5 weeks	Reduced fasting plasma glucose (FPG) and increased insulin levels, while improving lipid profiles by decreasing concentrations of triglycerides and low-density lipoprotein. Also, protected against renal damage by decreasing pro-inflammatory markers such as TNF-α, IL-1β, IL-6, cyclooxygenase-2 and inducible nitric oxide synthase (iNOS) in renal tissue
Jaja-Chimedza et al. (2018)	HFD-induced obese C57Bl/6 J mice treated with *Moringa oleifera* seed, containing 0.54 and 0.73% of extract supplemented in diet for 12 weeks	Reduced body weight, decreased adiposity, improved glucose tolerance, decreased inflammatory gene expression, and increased antioxidant gene expression. Specific, inflammatory genes that were decreased included IL-1β, IL-6 and TNF-α, while oxidative genes improved included iNOS and NADPH dehydrogenase [quinone] 1 (NQO1), in some of the tissues (liver, jejunum, ileum and colon)
Chin et al. (2019)	HFD and streptozotocin-induced diabetes in Sprague Dawley rats treated with 0.5% standardized aqueous *Moringa oleifera* leaf extract-loaded hydrocolloid film for 3 weeks	Significantly improved wound healing, and this was in part by effective modulation of pro-inflammatory markers and growth factors including TNF-α, IL-6, MCP-1, vascular endothelial growth factor, epidermal growth factor in the wound site
Mohamed et al. (2019)	HFD-induced insulin resistant in Sprague Dawley rats treated with 300 mg/kg *Moringa oleifera* aqueous extract for 4 weeks	Reversed hepatic insulin insensitivity and this was linked to up-regulation of genes involved in insulin receptors and glucose uptake such as insulin receptor, insulin receptor substrate-1 and glucose transporter (GLUT)4. Also improved hepatic antioxidants like catalase (CAT) and superoxide dismutase (SOD), while decreasing level of lipid peroxidation, the malonaldehyde (MDA) content
El-Shehawi et al. (2021)	HFD-induced obese Wistar rats treated with 300 mg/kg *Moringa oleifera* leaf extract for 14 weeks	Reduced body weight and body fat mass, while also decreasing FPG, insulin, and leptin levels, while increased adiponectin. Consistently, treatment improved lipid profiles like serum total cholesterol, triacylglycerol, and low-density lipoprotein, while enhancing hepatic antioxidant enzymes such as SOD, CAT. Lipid peroxidation (MDA) and some pro-inflammatory markers like nuclear factor kappa β (NF-κβ)- P65 were decreased

## Safety and the Toxicity Profile of *Moringa oleifera*

It is currently acknowledged that a large population of people depend on medicinal plants to treat different diseases, which mainly due to ancestral knowledge (Palhares et al., 2015; Muvhulawa et al., 2022). Thus, the general interest in the use of medicinal plants to cure various disease conditions has grown over the years (Rakotoarivelo et al., 2015; Michel et al., 2020). Therefore, it is important for plants to be evaluated for their toxicity to know how safe they are for human use. Accumulative research shows that *Moringa oleifera* exhibits a lot of important biological properties such as antioxidant, anti-inflammation, anti-hyperglycemic properties over the past years proving that it is a good plant to use as an alternative therapeutic for diabetes (Omodanisi et al., 2017b; Gothai et al., 2017; Paula et al., 2017; Abd et al., 2020; Xiong et al., 2021). *In vitro* and *in vivo* studies that have been conducted to show that this plant has no lethal dose and is safe to use. Indeed, work by Villarruel-Lòpez and others showed that the use of *Moringa oleifera,* at different doses ranging from 100 to 500 mg/kg, is not toxic in rats (Villarruel-López et al., 2018). Albrahim and Binobead also used rats to show that *Moringa oleifera* alleviates vetsin-induced cytotoxicity, as measured by alterations in liver functions, oxidative stress, DNA damage, and liver injury (Albrahim and Binobead, 2018a). Reviewed evidence from Asare and co-workers revealed that *Moringa oleifera* is genotoxic at supra-supplementation levels of 3,000 mg/kg body weight, with intake mostly considered is safe at levels ≤1,000 mg/kg (body weight) (Asare et al., 2012). However, other studies have indicated that although available literature is very promising (Awodele et al., 2012; Ajibade et al., 2013; Stohs and Hartman, 2015; Patriota et al., 2020; Siddiqui et al., 2021; Teshome et al., 2021), additional clinical (human) to accomplish standardized extracts of this plant.

## Clinical Translation

Preclinical studies are important to understand the pathophysiological mechanisms implicated in disease development, and this aspect remains essential to explore the therapeutic potential of plant extracts and their derivative compounds (Bonjour et al., 1999; Steinmetz and Spack, 2009). Whereas screening plant extracts and their biological compounds using preclinical models has become a routine procedure to determine effective doses, pharmacokinetic profile and evaluate relevant toxicological aspects before commencement of clinical trials (Bonjour et al., 1999; Steinmetz and Spack, 2009). Although accumulative literature supports the beneficial effects of plant extracts and naturally derived compounds against diabetes (Hung et al., 2012; Jugran et al., 2021), persistent setbacks have been the limited number of studies entering clinical trial phase, which is a vital component in drug development. Likewise, there has been limited number of studies on the antidiabetic properties of *Moringa oleifera* extracts. At present, only a few randomized controlled trials have been published on the antidiabetic potential (Table 3) of *Moringa oleifera*. In 2016, Anthanont and co-workers showed that capsules of *Moringa oleifera* leaf powder (at a dose of 4 g), taken after an overnight fast and every 2 weeks, could significantly increase insulin secretion in healthy subjects (Anthanont et al., 2016). Leone and others demonstrated that randomly giving Saharawi people with diabetes a traditional meal supplemented with 20 g leaf powder of *Moringa oleifera* on two different days could improve postprandial blood glucose response when compared to nondiabetic controls (Leone et al., 2018). Dixit and co-workers (Dixit et al., 2018) reported that intake of extracts of *Moringa oleifera* (LI85008F) at 900 mg/day (combined with modest calorie restriction and physical activity) for 16 weeks could reduce waist and hip circumferences, and improved lipid profiles in overweight participants. Also, this was relevant to reduced low-density lipoprotein (LDL) cholesterol decreased, while high-density lipoprotein (HDL) cholesterol increased, resulting in a significantly improved LDL/HDL ratio. While Gómez-Martínez and co-workers reported that giving subjects with prediabetes six daily capsules of *Moringa oleifera* leaf powder (2,400 mg/day) improved fasting blood glucose and glycated hemoglobin (HbA1c) when compared to the controls. Recently, Díaz-Prieto et al., demonstrated that consumption of 6 × 400 mg capsule/day of *Moringa oleifera* dry leaf powder for 12 weeks indicated that plasma tumor necrosis factor alpha (TNF-α) was a significant predictor of the subject’s glycated hemoglobin (HbA1c) response in subjects with prediabetes (Díaz-Prieto et al., 2022). These results are consistent with some preclinical findings (Jaja-Chimedza et al., 2018; Mohamed et al., 2019), indicating that *Moringa oleifera* leaf extracts might be useful complications identified during the early development of T2D. Nonetheless, despite accumulative literature, as reviewed elsewhere (Ba et al., 2020; Watanabe et al., 2021), indicating that this plant might present with important antidiabetic properties, more needs to be done to confirm these in clinical settings. It remains crucial, to evaluate whether *Moringa oleifera* leaf extracts can prominent biomarkers of oxidative stress and inflammation, to verify preclinical findings.

**TABLE 3:** An overview of clinical studies reporting on the antidiabetic properties of *Moringa oleifera*.

Author, year	Country	Study population	Dose and intervention period	Clinical outcome
Anthanont et al. (2016)	Thailand	Healthy subjects (*n* = 10)	Received an oral dose of *Moringa oleifera* at increasing dosages of 0, 1, 2, and 4 g, and monitored for 0.5, 1, 1.5, 2, 4, and 6 h	Improved baseline insulin levels, but did not affect blood glucose concentrations
Taweerutchana et al. (2017)	Thailand	Therapy-naïve type 2 diabetes patients (*n* = 32)	Received receive either 8 g per day of *Moringa oleifera* leaf capsules for 4 weeks	No effect on blood glucose levels, although non-significantly improved blood pressure
Dixit et al. (2018)	India	Overweight participants (*n* = 70)	Received combined extracts of *Moringa oleifera* (LI85008F) at 900 mg/day (combined with modest calorie restriction and physical activity) for 16 weeks	Reduced waist and hip circumferences, and improved lipid profiles. Also, reduced low-density lipoprotein (LDL) cholesterol decreased, while high-density lipoprotein (HDL) cholesterol increased, resulting in a significantly improved LDL/HDL ratio
Leone et al. (2018)	Italy	Subjects with type 2 diabetes (*n* = 17)	Received, on 2 different days, a traditional meal supplemented with 20 g of *Moringa oleifera* leaf powder	Improved blood glucose control
Sissoko et al. (2020)	Mali	Subjects with type 2 diabetes (*n* = 35)	Received n 1 and 2 g respectively, of *Moringa oleifera* leaf powder, 30 min after eating the white bread and were monitored for up to 180 min	Reduced post-prandial glycaemia in diabetic patients
Gómez-Martínez et al. (2021)	Spain	Subjects with prediabetes (*n* = 34)	Received six daily capsules of *Moringa oleifera* dry leaf powder (2,400 mg/day) for 12 weeks	Improved fasting blood glucose (FBG) and glycated hemoglobin (HbA1c). However, did not affect microbiota, hepatic and renal function markers or the appetite-controlling hormones measured
Díaz-Prieto et al. (2022)	Spain	Subjects with prediabetes (*n* = 31)	Received consumed 6 × 400 mg capsule/day of *Moringa oleifera* dry leaf powder for 12 weeks	Plasma tumor necrosis factor alpha (TNF-α) was a significant predictor of the subject’s HbA1c response (improvement YES/NO; 77% correct classification) in the *Moringa oleifera* group

## Summary and Future Perspective

The swift prevalence of diabetes warrants urgent investigation into novel therapies to protect and better manage this chronic medical condition (International Diabetes Federation, 2021). Metformin and insulin, which are commonly used antidiabetic therapies, have certainly prolonged the lives of patients with diabetes (Joya-Galeana et al., 2011; Foretz et al., 2014; Bailey, 2017). Correspondingly, other effective interventions like physical exercise and caloric striction can be used to manage diabetes (Nyawo et al., 2021; Shakoor et al., 2021; Mthembu et al., 2022), however only a few individuals can constantly adhere to such strenuous interventions. Besides lowering glucose or improving insulin sensitivity, it has become imperative to uncover therapies that can target the amelioration of both oxidative stress and inflammation, as the prime dysregulations implicated in the pathogenesis of diabetes (Vikram et al., 2014; Mahlangu et al., 2020; Mokgalaboni et al., 2021b). This also explains the surge in research investigating the biological properties of nutritional plant sources like rooibos (*Aspalathus linearis*) and broccoli (*Brassica oleracea var. italica*) with abundant antioxidants properties in combating metabolic complications like oxidative stress and inflammation (Hwang and Lim, 2014; Dludla et al., 2017a; Orlando et al., 2022).

Plants have been studied for their therapeutic properties and they are also cheap, easily accessible and safer than synthetic conventional drugs (Ahmad et al., 2019). There is growing evidence that plants not only serve as a food source but as medicine, nutraceuticals and so forth (Alegbeleye, 2018). Also, they are a body of polyphenols, vitamins, flavonoids, alkaloids and other important phytochemicals. *Moringa oleifera* has been proven in a number of studies to alleviate insulin resistance by activating the insulin-independent pathway PI3K/AKT and also through AMPK pathway in the skeletal muscle and it can also improve skeletal muscle oxidative metabolism through the NAD-dependent deacetylase (SIRT1)-PPARα pathway and also through improving fatty acid peroxidation (Bao et al., 2020; Duranti et al., 2021).

**FIGURE 3:**
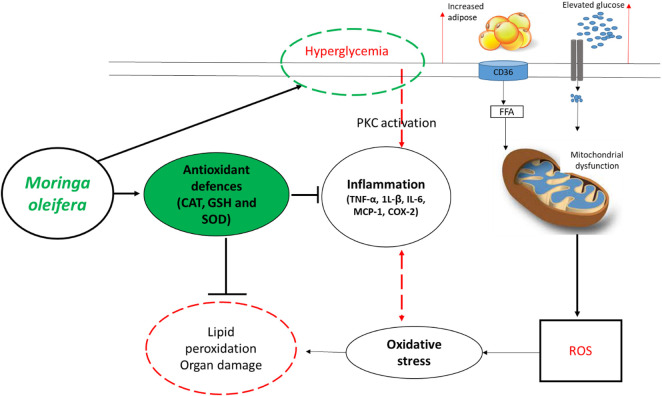
An overview of therapeutic mechanisms associated with the ameliorative effects of *Moringa oleifera* extracts in preclinical (animal) models of diabetes. Briefly, overwhelming evidence supports the beneficial effects of this plant in enhancing intracellular antioxidants such as catalase (CAT), glutathione (GSH) and superoxide dismutase (SOD) to block the detrimental effects reactive oxygen species (ROS), lipid peroxidation and organ damage. This is in part by also improving glucose control (hyperglycemia) and reducing prominent pro-inflammatory markers like tumor necrosis factor-alpha (TNF-α), interleukin (1L)-β, IL-6, monocyte chemoattractant protein-1 (MCP-1) and COX-2-cyclooxygenase-2 (COX-2). Abbreviations: CD36-cluster of differentiation 36; FFA-free fatty acid; PKC-protein kinase C. Indicators: red lines-detrimental effects, bold lines/green lines-protective effects of *Moringa oleifera* extracts.

In fact, overwhelming evidence summarized in this review supports the beneficial effects of *Moringa oleifera* in improving blood glucose levels, lipid profiles and insulin sensitivity, in addition to protecting against hepatic or nephrotic damage in preclinical (animal) models of T1D/T2D (Table 1 and 2). Interestingly, these extracts show enhanced effects in strengthening intracellular antioxidants like CAT, SOD, GSH, and GST to lower lipid peroxidation products MDA/TBARS, and reduce prominent pro-inflammatory markers like TNF-α, 1L-β, IL-6, MCP-1, COX-2, and nitric oxide synthase in these animal models. Figure 3 summarizes some therapeutic effects in protecting against oxidative stress and inflammation associated with *Moringa oleifera* extracts in preclinical models of diabetes. Furthermore, the current literature review indicates the common use of leaf extracts of *Moringa oleifera*, within a range 100–300 mg/kg, from initial treatment duration of 2 weeks up until 8 weeks (Tables 1, 2). This further sets a platform for future research (which is currently limited) directed at developing *Moringa oleifera* as a functional food to manage diabetes mellitus. Importantly, additional clinical trials are necessary to evaluate whether *Moringa oleifera* leaf extracts can prominent biomarkers of oxidative stress and inflammation, to verify preclinical findings.

## Glossary

AMPK-AMP: activated protein kinase
AKT: protein kinase B
ACOX1: peroxisomal acyl-CoA oxidase 1
BCL-2: B-cell lymphoma 2
CAT: catalase
C/EBPα: enhancer-binding protein
COX-2: cyclooxygenase-2
GPx: glutathione reductase
GST: glutathione-S-transferase
GLUT4: glucose transporter 4
GSH: glutathione
HbA1C: glycated hemoglobin
HDL: high density lipoprotein
HO-1: heme oygenase-1
LDL: low density lipoprotein
NJK-c-Jun N: terminal kinases
MDA: malonaldehyde
iNOS: inducible nitric oxide synthase
INF-γ: interferon gamma
IL: interleukin
MAPK: mitogen-activated protein kinase
MCP-1: monocyte chemoattractant protein-1
NF-κβ: nuclear factor kappa β
T1D/T1D: type 1/2 diabetes mellitus
TNF-α: tumor necrosis factor alpha
TBARS: thiobarbituric acid reactive species
PDE-5: phosphodiesterase type 5
PKC: protein kinase C
PPARγ: peroxisome proliferator-activated receptor gamma
SIRT1: NAD-dependent deacetylase
SOD: superoxide dismutase
SREBP1c: sterol regulatory element-binding protein 1c
WHO: World Health Organization
VEGF: vascular endothelial growth factor
