# Corrigendum: A review on the antidiabetic properties of *Moringa oleifera* extracts: Focusing on oxidative stress and inflammation as main therapeutic targets

**DOI:** 10.3389/fphar.2023.1142410

**Published:** 2023-01-24

**Authors:** Fikile T. Mthiyane, Phiwayinkosi V. Dludla, Khanyisani Ziqubu, Sinenhlanhla X. H. Mthembu, Ndivhuwo Muvhulawa, Nokulunga Hlengwa, Bongani B. Nkambule, Sithandiwe E. Mazibuko-Mbeje

**Affiliations:** ^1^ Department of Biochemistry, North-West University, Mafikeng, South Africa; ^2^ Biomedical Research and Innovation Platform, South African Medical Research Council, Cape Town, South Africa; ^3^ Department of Biochemistry and Microbiology, University of Zululand, KwaDlangezwa, South Africa; ^4^ School of Laboratory Medicine and Medical Sciences, University of KwaZulu-Natal, Durban, South Africa

**Keywords:** diabetes complications, oxidative stress, inflammation, Moringa (*Moringa oleifera*), therapeutic targets

In the published article, there was an error in the Funding statement.

“This work was funded by the National Research Foundation (NRF) Thuthuka Programme grant 128296 to SM-M. The North-West University, South African Medical Research Council and the University of Zululand is also acknowledged. All the content expressed in this review is the official views of the authors and do not represent that of the North-West University. FM acknowledges funding by the NRF, Thuthuka grant UID 128296 linked to SM-M.”

The correct Funding statement appears below.

“This work was funded by the National Research Foundation (NRF) Thuthuka Programme grant 128296 to SM-M. Funding from North-West University and the University of Zululand is also acknowledged. The work reported herein was made possible through funding by the South African Medical Research Council (SAMRC) through its Division of Research of Capacity Development under the Early Investigators Programme from the South African National Treasury (funding number: HDID8682/MB2022/EIP052). The content hereof is the sole responsibility of the authors and do not necessarily represent the official views of the SAMRC. Also, all the content expressed in this review is the official views of the authors and do not represent that of the North-West University or the University of Zululand. FM acknowledges funding by the NRF, Thuthuka grant UID 128296 linked to SM-M.”

The authors apologize for this error and state that this does not change the scientific conclusions of the article in any way. The original article has been updated.

